# An Online Health Prevention Intervention for Youth with Addicted or Mentally Ill Parents: Experiences and Perspectives of Participants and Providers from a Randomized Controlled Trial

**DOI:** 10.2196/jmir.4817

**Published:** 2015-12-02

**Authors:** Marla Woolderink, Jill APM Bindels, Silvia MAA Evers, Aggie TG Paulus, Antoinette DI van Asselt, Onno CP van Schayck

**Affiliations:** ^1^Clinical Epidemiology and Technology AssessmentMaastricht University Medical CenterMaastrichtNetherlands; ^2^CAPHRI-School for Public Health and Primary CareMaastrichtNetherlands; ^3^Department of Health Services ResearchMaastricht UniversityMaastrichtNetherlands; ^4^Institute of Mental Health and AddictionTrimbos InstituutUtrechtNetherlands; ^5^Department of EpidemiologyUniversity Medical Center GroningenUniversity of GroningenGroningenNetherlands; ^6^Department of PharmacyUniversity of GroningenGroningenNetherlands; ^7^Department of General PracticeMaastricht UniversityMaastrichtNetherlands

**Keywords:** online-delivered course, process assessment, qualitative research, mental health, prevention, adolescents

## Abstract

**Background:**

Mental illnesses affect many people around the world, either directly or indirectly. Families of persons suffering from mental illness or addiction suffer too, especially their children. In the Netherlands, 864,000 parents meet the diagnostic criteria for a mental illness or addiction. Evidence shows that offspring of mentally ill or addicted parents are at risk for developing mental disorders or illnesses themselves. The Kopstoring course is an online 8-week group course with supervision by 2 trained psychologists or social workers, aimed to prevent behavioral and psychological problems for children (aged 16 to 25 years) of parents with mental health problems or addictions. The course addresses themes such as roles in the family and mastery skills. An online randomized controlled trial (RCT) was conducted to assess the effectiveness of the Kopstoring course.

**Objective:**

The aim was to gain knowledge about expectations, experiences, and perspectives of participants and providers of the online Kopstoring course.

**Methods:**

A process evaluation was performed to evaluate the online delivery of Kopstoring and the experiences and perspectives of participants and providers of Kopstoring. Interviews were performed with members from both groups. Participants were drawn from a sample from the Kopstoring RCT.

**Results:**

Thirteen participants and 4 providers were interviewed. Five main themes emerged from these interviews: background, the requirements for the intervention, experience with the intervention, technical aspects, and research aspects. Overall, participants and providers found the intervention to be valuable because it was online; therefore, protecting their anonymity was considered a key component. Most barriers existed in the technical sphere. Additional barriers existed with conducting the RCT, namely gathering informed consent and gathering parental consent in the case of minors.

**Conclusions:**

This study provides valuable insight into participants’ and providers’ experiences and expectations with the online preventive intervention Kopstoring. It also sheds light on the process of the online provision of Kopstoring and the accompanying RCT. The findings of this study may partly explain dropout rates when delivering online interventions. The change in the (financial) structure of the youth mental health care system in the Netherlands has financial implications for the delivery of prevention programs for youth. Lastly, there are few RCTs that assess the effectiveness and cost-effectiveness of online prevention programs in the field of (youth) mental health care and not many process evaluations of these programs exist. This hampers a good comparison between online interventions and the expectations and experiences of the participants and providers.

**Trial Registration:**

Nederlands Trial Register: NTR1982; http://www.trialregister.nl/trialreg/admin/rctview.asp?TC=1982 (Archived by WebCite® at http://www.webcitation.org/6d8xYDQbB)

## Introduction

Mental illnesses affect many people around the world, either directly or through another person. The prevalence of mental illnesses, such as depression, is high [1] and many among those who suffer from mental illness or addiction are parents. The families of persons suffering from mental illness experience a degree of burden too [2,3]. Results of a Canadian survey show that 1 in every 10 children lives with a parent with a psychiatric disorder and 1 in every 6 children lives in a household with at least 1 person affected by a psychiatric disorder [4]. In these situations, mental illness is not only a problem for the patients, but also for their family and their children in particular. Various studies report that offspring of mentally ill/addicted parents are regarded at risk for developing mental illness (eg, depression and anxiety disorders) themselves [5-8].

Although preventive interventions for children of parents with mental illness or addiction are scarce, some interventions have been developed for this vulnerable group [9,10]. Most of these interventions are intended to be performed face-to-face and only a few are developed for online use. However, given the nature of the target group (eg, including minors, being at risk) and the problem being addressed, a face-to-face intervention is associated with numerous challenges regarding recruitment and inclusion. National Dutch data show that with current face-to-face interventions, the target population was not reached sufficiently and information did not find its way to the population [11]. The youth that were reached valued their anonymity and privacy, which makes face-to-face interventions less appealing. Therefore, online interventions seem to be a worthy alternative.

In the Netherlands, 864,000 parents meet the diagnostic criteria of a mental illness or addiction [12,13]. An online preventive course (Kopstoring) for children of these parents was developed. Kopstoring is one of the few online interventions for children of parents with mental illness or addiction disorders. The course is based on evidence-based theories and a face-to-face course developed for the same population. The Dutch Kopstoring course is designed for adolescents from ages 16 to 25 years. A pilot study assessing the effects of the course showed Kopstoring to be effective in improving participants’ coping and mastery mechanisms [14].

The objective of this study is to gain knowledge about expectations, experiences, and perspectives of participants and providers of the online Kopstoring course. The research questions were how was the process of the delivery of the online Kopstoring course perceived by Kopstoring participants and providers and what were their expectations and experiences with this course?

## Methods

A process evaluation was performed to evaluate the online delivery of Kopstoring and the experiences and perspectives of Kopstoring participants and Kopstoring providers. Interviews were performed with both groups. This section first describes the Kopstoring course and the accompanying randomized controlled trial (RCT; trial registration: NTR1982) [15] and subsequently the methods used in this process evaluation.

### Intervention

The Kopstoring course aimed to prevent behavioral and psychological problems in offspring at risk and was offered to adolescents from ages 16 to 25 years. The Kopstoring course was an online 8-week group course with supervision by 2 trained psychologists or social workers from a participating mental health institution in the Netherlands. Every week a different theme was discussed and participants were expected to prepare for the weekly meetings by doing homework. The course had a preventive nature; therefore, adolescents were screened to ensure that they were not diagnosed with an illness as classified by the *Diagnostic and Statistical Manual of Mental Disorders*, Fourth Edition (*DSM-IV*) diagnoses. Screening was executed by the mental health institutions. In addition, participants needed to have access to a computer with an Internet connection and be able to participate weekly.

Alongside the process evaluation described in this paper, a RCT was conducted. The aim of the RCT was to examine the effectiveness and cost-effectiveness of the Kopstoring course. Participants were randomly allocated either to immediate enrollment in the Kopstoring course (intervention group) or enrollment after a 6-month waiting list (control group). Because the course was completely digitalized, the recruitment was done mainly, but not exclusively, through online recruitment, including banners, Facebook advertisements, links to the website, etc. In addition, articles were published in national and regional magazines and newspapers, school visits were performed, and an interview was broadcasted on a radio station.

### Sample

In this process evaluation, 2 groups were included: participants (n=13) and providers (n=4) of the Kopstoring course.

The Kopstoring participants were selected from both the intervention and the control group of the RCT. Participants received an email in which they were invited to be interviewed. To select participants for the interviews from the trial pool, a maximum variation strategy was used to gather information from a sample with as much variation as possible to collect as many different perspectives [16]. This was done by looking at several characteristics (eg, trial arm, age, sex, online and written consent for the trial, dropout). See Table 1 for the characteristics of the Kopstoring participants who were interviewed. The interview sample is fairly comparable to the RCT sample.

**Table 1:** Characteristics of interview participants versus trial participants.

Characteristics		Interview participants, n (%) n=13	Trial sample, n (%) n=104
**Age** ^a^ **(years)**			
	16-17	3 (23)	20 (19.2)
	>18	10 (77)	84 (80.7)
**Sex**			
	Female	12 (92)	93 (89.4)
	Male	1 (8)	11 (10.6)
**Treatment group**			
	Intervention	8 (62)	55 (52.9)
	Waiting list control	5 (38)	49 (47.1)
**Adherence to Kopstoring course** ^b^			
	Completed	11 (85)	97 (93.3)
	Started but did not finish	2 (15)	7 (6.7)

^a^ Age at time of registration for the course.

^b^ To this point, data were checked up until 6 months after registration due to the pending follow-up assessments.

At the start of the project, 9 Dutch mental health institutions participated and each institution trained 2 professionals for the provision of the Kopstoring course. All planned Kopstoring courses provided during this study were provided by 7 professionals from 4 different mental health institutions. These providers were invited to participate in interviews. In total, 4 providers agreed to participate in an interview.

The providers of the Kopstoring course were all female and approximately 30 years of age; all had a Master’s degree and had experience working in this field for 5 to 7 years.

### Data Collection

Data were collected through semistructured individual interviews with a list of topics to be discussed (Textbox 1). Interviews were held between November 2014 and February 2015. The topic list was made by the research team in collaboration with the national coordinator of Kopstoring and the team of course providers.

**Interview topic list for participants.**

Contextual information

- Situation analyses
- Context analyses

Website Kopstoring

- Views about website
- Information delivery
- Logistics website

Effects of the course

- Views about the course
- Anticipated effects
- Experienced effects before, during, and after the course
- Barriers and success factors for completing the course

Process and content-related aspects of the course

- Components (themes) of the course
- Tailor-made health care
- Technical aspects of delivery online

Research

- Understanding study aspect
- Motivation
- Experience

Anonymity was very important for offspring of parents with mental illness or addiction problems. Therefore, interviews were conducted over the phone. Participants decided the time of the interview so they could be sure they were able to talk freely. Interviews with the providers were also conducted over the phone, but due to time constraints not because of anonymity. Interviews were held in Dutch. Textbox 2 displays the topic list for the providers.

### Analysis

The interviews were audiotaped and transcribed and identifiable information was removed to ensure anonymity. The interviews were analyzed by using inductive qualitative content analysis, specifically conventional content analyses [17]. This method helped provide an in-depth understanding about underlying perspectives and qualitative methods are inductive and reflexive and it allowed the use of quotes [18]. As a first step, the interviews were read by 2 researchers separately to identify emerging themes and subthemes and then labels were attached to the parts related to these themes. Secondly, new themes were added to existing themes and labeled accordingly. After the 2 researchers reached consensus, the interview data were clustered into themes and subthemes. Finally, citations of the interviewees were identified per theme and visualized in a data matrix. After approximately 13 interviews, no new information emerged from the interviews with the participants.

## Results

Five main themes emerged from the interviews: (1) background, (2) the requirements for the intervention, (3) experience with the intervention, (4) technical aspects, and (5) research aspects. In this section, each theme and its subthemes will be discussed from the participants’ and the providers’ perspectives.

### Background

For the participants, the background mainly related to the motivation and reason for participation, the route to registration, and expectations of the online course. The providers’ background related to their experiences with the provision of similar face-to-face courses and online interventions.

**Interview topic list for providers.**

Contextual information

- Mental health institution
- Personal background
- General impression Kopstoring
- Financial situation and implications (Mental health institution)

Website Kopstoring

- Views about website
- Information delivery
- Logistics website

Provision, process, and content of the course

- Views about the content of course
- Advantages and disadvantages of online delivery
- Experiences with provision of Kopstoring
- Process from registration to allocation in group
- Barriers and success factors for provision of the course
- Technical aspects of delivery online

Research

- Motivation to participate
- Experience with the study

#### Participants

Participants from the Kopstoring course generally had 2 routes to arrive at the point of registration. Analyses showed that it was either a slow, lingering process in which the person already had the intention to change the situation for some time and was looking for a suitable way to address their needs or there was an acute situation which forced them to seek help right away. The following is an example of an acute situation that led to an immediate online registration:

There was a real occasion leading to why I registered. It was September last year and my mother had a psychosis...and she attacked me that night.

Participant 12

A respondent for whom the situation was ongoing long before registration explained:

I had a difficult time dealing with the situation and with the fact that my brother was placed into care (out of house placement). Well, I really could not handle it well, so they advised me to register for the course.

Participant 4

There was no difference in results reported by participants who registered under pressure of an acute event or those who took their time to register for the course.

For both situations, there appeared to be several facilitators, for example, a psychologist, school mental health worker, or a family member pointing out the online course or participants who found the course through an Internet search. Despite the different problems and family situations of participants (eg, one person had an addicted mom, another had a mentally ill father, and both parents were mentally ill for a third person), the consequences, questions, and problems they were confronted with were very similar.

Motivations to participate could be divided into 4 categories: (1) sharing experiences with persons in the same situation, (2) learning how to cope with ill parents, (3) learning how to cope with their own problems, and (4) learning about mental illness or addiction:

I hope it will be comforting for me to talk about my experiences with peers who went through the same experience.

Participant 11

Most of the time, problems were not discussed with family members and friends. This explained their need for sharing with others who had been through the same experience. One participant explained this as:

In a certain way, it provoked a sense of relief learning that other people were actually going through the exact same experience.

Participant 4

All participants had easy access to the website and experienced no problems with the registration process. During this process, all prospective participants were asked about their expectations of the online course. The answers were concise for the most part and participants had clear expectations regarding the content and the anticipated effect of the course.

When asked about their goals, participants reported they expected to learn and understand more about their parent’s illness or addiction, to learn how to cope with the illness or addiction, and to learn how to improve the situation at home and decrease problems themselves.

One respondent explained that she hoped to find out how the situation got this “extreme” and to learn to deal with her mom so that they could improve their relationship and the situation. In accordance, other participants explained:

I expect this to be very helpful, mainly that I have more understanding about the cause of the symptoms and how to deal better with my mother. I hope to learn ways and also to detach myself from my mother.

Participant 10

Prospective participants, aged 16 to 25 years, had well-defined expectations of the online delivery and effects of the Kopstoring course.

#### Providers

Providers’ general impression of the online delivery of Kopstoring was positive:

It is just a very good program that does not require any change. That is, of course, very important.

Provider 1

In some cases, the Kopstoring manual was considered theoretically so well written that providers used the same manual for face-to-face groups for children of mentally ill or addicted parents:

Yes, I find that very good [the course manual/protocol]. I even use it as the manual in the face-to-face Kop-groups. This is because I consider it to be a very pleasant way how subjects are being discussed, which themes will be covered, like cognitive behavior therapy.

Provider 3

### Requirements

There are some requirements when providing online interventions. Firstly, participants and providers needed a computer connected to the Internet, log-in codes, and some privacy. For the providers, a budget was necessary to provide the Kopstoring course. Some barriers were encountered at the organizational level and regarding the financial structure.

#### Participants

In general, there were no barriers encountered to meet the requirements; however, one of the participants mentioned that when moving house she did not have access to the Internet, which made it impossible to log on to that session.

A second person explained that it was not always easy to find enough privacy in the house because there were always people around who did not know he was participating in the course:

The only thing that was difficult was finding a place to separate myself from others, and just having a moment for myself. That was difficult.

Participant 12

#### Providers

Providers needed support from their managers within the mental health institution and adequate finances to provide the online course because online mental health interventions are not paid from public funds. In addition, providers of online anonymous interventions face the situation that costs will not be reimbursed by insurers due to the fact they cannot provide social security numbers or other personalized details. It is up to the management of a mental health institution to decide whether or not it is feasible to add Kopstoring in their portfolio. In addition, the financial situation and structure of mental health care divisions for minors (up to age 18 years) changed during the course of the RCT. In short, municipalities became responsible for the policy and execution of budgeting of prevention interventions in youth mental health care. This shift had tremendous consequences for the delivery of mental health care interventions for youth up to age 18 years. In some institutions, prevention and youth departments were declared redundant and, consequently, the institutions withdrew their consent to provide the Kopstoring course. There were many problems encountered with finding funding to provide the Kopstoring course. One provider explained:

Health insurers are not paying for delivery of Kopstoring because it is provided anonymously and a health insurer only wants to reimburse when they have all details from the client. So that means you have to provide them with a health insurer registration number and social security number, everything, and we do not ask these details when providing Kopstoring because we want it to be anonymous. So the only remaining source is the municipality and...naturally the municipality actually only wants to pay for inhabitants of that municipality.

Provider 1

Initially, the costs of the courses were reimbursed by additional funding obtained by the research team. This meant that when the study period ended, reimbursement of the courses also came to an end. One provider explained that once the research team did not fund provision of the courses anymore, their mental health institution stopped providing the Kopstoring course.

### Experiences with the Course

#### Participants

Participants described many different effects on their daily life and their problems. The first and most emphasized effect of the course was peer contact. Speaking with youth in the same situation made participants feel less alone, relieved, and less guilty in some cases. The recognition of situations, problems, and decisions became something they could share with peers:

My friends did not understand me. I have tried to explain, but then they would just say “awhhh it will be fine” and that was so nice to...with peers who might have a slightly different situation maybe, but that they also felt lonely and that you...share the same and have compassion for one another.

Participant 5

A second component that was considered very effective was the psychoeducative part of the course. Learning about the illness or addiction of the parent gave insight into the behavior of the parent:

I noticed that I experienced more peace with the fact that she has a drinking problem. It is the way it is and that will not change anymore.

Participant 3

Furthermore, the participants learned tips and tricks on how to cope with the behavior and problem of the parent, which led to accepting the parent’s problem and more peace in the family in some cases:

Yes, I argued with my father pretty often if he had something going on. They said I should actually try to reduce these moments and I am able to do that now.

Participant 1

Participants also reflected on the content of the Kopstoring course and all themes were deemed important for the course to be effective. Almost all interviewees pointed out that the “rate your week” component, which kicked off every session, was extremely valuable to them. “Rate your week” was a simple but effective way to share experiences about the past week and a platform for questions and peer contact. A respondent explained the working mechanism of “rate your week”:

Rate your week was very interesting for me. For your self-reflection on whether or not the week went well and that you were able to look back later to see how it was in the beginning of the course and how am I doing now? I really liked that; what are the positive things that make you also feel very positive instead of focusing on the negative things.

Participant 9

However, one of the sessions in which the educational component was key was mentioned to be a bit repetitive.

Experiences with the online program also translated into barriers. Barriers that existed were lack of time to discuss the homework assignments, some participants mentioned the course focused too much on the younger participants (students), and a couple of participants mentioned the homework was complicated.

Facilitating factors to adhere to the intervention and study were also mentioned. The online delivery of the intervention was mentioned as a major facilitating factor to start and finish the course. Firstly, online delivery was found to be convenient and ideal for participation in a safe and self-chosen environment. Secondly, online delivery protected the anonymity and privacy of the participant, which encouraged the participants to be more open:

Openness, yes...because it is online you do not have the feeling that everyone was looking at you. Then you can just write and maybe if you had to cry or so...no one was able to see that.

Participant 8

An often-mentioned stimulating factor was the attitude of the provider. Most of them were easy to access and always available (by email) to answer questions and monitor the participant:

I noticed that whenever the trainer tells us that she is still available to answer questions after the end of the course, or emails or these kind of things, that felt incredibly nice, that someone is still there who takes time, yeah where you can lean on. So that I consider to be very pleasant.

Participant 6

Only one participant mentioned that the attitude of the provider was not meeting her expectations. This participant stopped participating in the course after session 3 and was not included in the RCT.

#### Providers

All providers were satisfied with the content and agreed that all the important concepts were covered. The most important aspect was considered to be the online delivery, which ensured anonymity for the participant:

Within the Kopstoring course, they [participants] can, of course, tell their story very anonymously. Nobody knows that you participate in the group and what is bothering you. That is a huge advantage; that it becomes easily accessible for youngsters, but that they nevertheless can benefit and become more aware of what is going on and get answers to their questions.

Provider 2

For the content, the most important part was considered to be the exchange of experiences during “rate your week” at the beginning of each session.

All providers were asked several questions to check adherence to the protocol. They indicated they followed protocol except for one rule: the protocol described delivery of the course should be done by 2 professionals together. In practice, all providers delivered the course individually due to cost reductions. This was, however, not considered to be a barrier because providing the course for a group of up to 6 participants was highly manageable for one provider. There were some (technical) barriers experienced for the online delivery, but for providers the main barriers were experienced in the financial administrative field.

Positive factors were described as the feasibility of the online delivery and the possibility to deliver the course from home, the interactive group process, and the growing number of participants:

That every time again I am so surprised how close a group can become online and that as quick as in the first session they are already so open. And that is due to the anonymity that participants are just so open and what they think or experience...Yes, I think that this is very special and that stimulates me to provide the course over and over again and just getting back from them that they appreciate being heard.

Provider 1

### Technical Aspects

The technical component was found to be extremely important by both the participants and providers of the Kopstoring course. Not only were the technical aspects (eg, the website or the chat box) considered positive factors, the same technical aspects were mentioned as barriers for participating in or providing the course. Almost every interviewee mentioned technical problems of some degree (from having a slow system to being thrown out of the chat box) and providers also mentioned these disturbances interrupting the courses. However, there were no major incidents mentioned that fully hindered participation in the long run.

#### Participants and Providers

The delivery of Kopstoring online was considered very positive; however, it seemed to also cause problems. Online delivery can be a double-edged sword; the convenience of the online aspect can be a pitfall at the same time.

Technical aspects were the design of the website and the chat box. Both were considered well designed and suitable for the target population. The website was described as complete, clear, colorful, and cheerful, which for participants is important. Providers shared this opinion about the website:

Just by clicking on the website, I consider it quite clear...I think it is convenient when registering for the course that the data when courses are starting is visible.

Provider 2

I considered it [chat box] very well done that different persons were indicated with different colors, so you were able to see who...to increase visibility.

Participant 3

### Barriers

The technical problems described by both groups ranged from technical hiccups to some more prominent problems, such as not being able to log on to group sessions or being locked out by the system.

Minor technical problems involved a slow system, not being able to see when someone was typing, unable to see homework assignments on the screen, and double messaging occurring. These problems were mentioned, but were interpreted as minor problems and a consequence of an online working environment:

Sometimes it took like a minute or so before the text would be displayed or then it got stuck or we were removed from the chat box. Yeah every now and then we would struggle a bit.

Participant 12

Providers also described experiencing the same minor problems. The technical platform and responsibility related to the technical aspects were more numerous for the providers. They were responsible for all requirements to be met even before groups started online sessions.

### Research Aspects

Participants were confronted with aspects such as a 6-month waiting list, randomization, extended follow-up, and questionnaires. It appeared that most participants understood there was a study linked to the Kopstoring course, but none of them could describe what the consequences were for them; regardless, patient information sheets were given to them by email, mail, and online:

I did know there were more groups where you could be allocated to, but I did not know that there was a chance you would have to wait half a year.

Participant 12

Participants who were allocated to the waiting list believed they had to wait because the group was full. This explained why most participants expressed no strong negative experiences toward the research components. In some cases, the waiting list was experienced as problematic, although most participants accepted the waiting period:

Yes, there was one group and I was hoping I could start right away, but unfortunately no. I had to wait half a year. That was really annoying. I needed help at that particular moment.

Participant 8

Most of the participants indicated altruism as the main reason for participating in the study, although others participated because “it is part of the course”:

I just hope that there are more young adults who get this opportunity to participate in this kind of course...that here is research, because yeah I feel that there is too little for Kopp? (children of mentally ill or addicted parents, for those groups).

Participant 9

Participants provided feedback on the length of the questionnaires and some technical problems related to not being able to open links or links expiring due to waiting too long to fill out the questionnaire. From the interviewed Kopstoring participants, 2 persons had incomplete data; when asked why, there was no specific reason, but they said they forgot. In addition, some of the respondents mentioned the phrasing of some of the questions. They disliked the questions because they were too focused on the younger participants (students) living with their parent(s).

During the interviews, a couple reasons for the poor response rates were mentioned: laziness or forgetfulness and problems with parental informed consent in case of a minor.

Participants were asked to give online consent and written consent sent by post. Only one participant did not send back the informed consent papers and, therefore, was not a participant in the RCT. She explained that she forgot to send the forms back, whereas the other participants had clear motivations for participation in the study. Some minors sent back their informed consent papers, but not those of their parents, stating they did not wish their parents to know let alone sign a consent paper for participation in the study. Some minors found a way to let their parents sign.

#### Providers

For providers, the research aspects were proper barriers. The back office and technical interface changed so much with the migration to the study environment that the coordinator was forced to invest time into teaching herself the new system. Providers also pointed out that the inclusion of participants in the study would have been smoother if the research team had listened to their advice concerning some of the requirements for study inclusion:

What I consider to be a real pity is that not enough weight is given to the advice of the professionals from the mental health institutions. ...Yeah, youngsters score incredibly high...if you are shocked by that as a researcher...It doesn’t surprise or shock me anymore because I know that they score high, and I think it is very important to just reach out to these youngsters for participation in Kopstoring and motivate them to seek help.

Provider 1

One other barrier mentioned was the frustration they had to deal with when a person was allocated to the waiting list control group. Providers explained, “It is the way it is when doing research but it remained sad you had to disappoint a person who needs help right there and right then.”

Every 3 weeks, a new group was started. In addition, due to the allocation of participants to the waiting list, the number of people in the groups was reduced. Starting with smaller groups was considered a disadvantage because it was difficult to reach the full potential of the course.

Regardless of these barriers, providers were determined to reach the target number of participants for inclusion in the RCT. Their motivation was based on several stimulating factors. One provider explained that her opinion about the value of the product made her enthusiastic to provide the course and help with inclusion in the study:

I think you are enthusiastic if you see the value of research. That will lead to results and, surely, I hope the results are good. You also notice that participants have very positive experiences and so you feel you are working/providing a good product, so I think it matters a lot and the fact that it is studied, I only cheer for that.

Provider 4

Also, the interest and engagement of the researcher played a role in the delivery of the Kopstoring course and the willingness to help:

I enjoyed that you (researcher) were present at all meetings and gave an update on how the situation was and, yes, then we had an idea of what the situation was and that is what you are working towards.

Provider 2

Providers explained there were barriers; despite these, they were able to work within the study parameters.

## Discussion

### Principal Results

To our knowledge, this study is the first evaluation of the experience of providers and participants with an online-delivered prevention course for offspring at risk. Therefore, this study differentiates itself from existing international literature and provides new information. The few process evaluations performed to assess experiences with online programs focus on online treatment, programs for somatic diseases, or and/or an adult patient population. The findings of this study give insight into the experiences of participants and providers of an online prevention course called Kopstoring. It sheds some light on experience with as well as barriers and facilitating factors of online delivery. It elaborates on the expectations and experiences of both participants and providers. Analyses showed similar experiences for the 2 groups despite their different perspectives.

The main lesson learned from participants lies in their assessment of the course content and the barriers and facilitating factors for participating and adhering to an online course. The online aspect and anonymity proved to be important as well as their autonomy to decide to participate without interference from anyone else. In the Netherlands and some other countries, minors (participants younger than 18 years) need to provide the research team with written consent and their parent’s consent for participating in a scientific study. This ignores the fact that minors can receive treatment (which is being assessed) of any kind from the age of 16 years without parental consent. Youth are considered capable of making an informed decision about treatment; however, for a scientific study we doubt their ability to make an independent and informed choice [19]. This subject was brought up even by participants older than 18 years who said that if they had to provide parental consent, they would probably not have participated. They explained that we cannot expect them to ask their parents for consent when they are the root of their problems. There is a strong need to rethink the policy concerning consent in the case of interventions for vulnerable populations and interventions with a high level of anonymity (mostly online interventions). This statement endorses the debate in the literature questioning when a minor should be considered capable to give informed consent and therefore protect his/her anonymity [20,21]. Despite the differences in consent procedure for minors, no substantial differences between minors and participants older than 18 years are reported in this study.

The lesson learned from the providers of the Kopstoring course lies partly in their professional assessment of the content of the course, but mainly in the experience with providing online courses and the barriers and facilitating factors to provide the Kopstoring course. Analyses showed that providers of online interventions in RCTs might feel ignored and may experience a gap between the research team and providers, even though the provider has many years of experience with providing online interventions in this target population. This implies that there is a need for closer collaboration with providers, and perhaps even with the target population, when designing such interventions and accompanying studies [22,23]. Collaborating with stakeholders could have led to other research questions, methods, and the use of other questionnaires more suitable for the target population.

The current situation in the Netherlands for children in need of mental health care is unsettling. This study could not have taken place in a more inconvenient time and political setting than it actually did. In the same period the RCT was running, political decisions forced youth mental health care out of the hands of mental health institutions and made it subsequently a part of the local municipalities. Even if the results of cost-effectiveness studies, such as the Kopstoring RCT, show positive results, online interventions may not be provided due to the complex financial structure and lack of responsible bodies to finance online interventions. This also shows that implementation and implementation research in the Netherlands, but very likely in other similar countries, is nearly impossible for these types of intervention.

### Limitations of the Study

There are several factors that could be considered to influence the findings of this study. The first is the number of interviews performed. One can question whether the small number of providers interviewed is sufficient to provide a complete overview of the ongoing issues. However, we do believe that providers who cooperated gave a lot of information about the Kopstoring course and the delivery of the course. We remained with only 9 providers who provided more than one course and were totally informed about every research detail. Therefore, we believe 4 providers were a good reflection of the 9 remaining providers and the group appeared to be homogeneous.

For the participants, there is a different reason for the low response rate (42 people were invited, 17 responded, and 13 were eventually interviewed; response rate: 13/42, 31%) for participating in this study. The target population appears to be extremely difficult to reach. As shown in the analyses, they wish their anonymity to be respected and feel “safe” in an online environment and not face-to-face or on the phone. In addition, a feeling of shame and guilt regarding their problems blocks them from sharing their experiences with a researcher. Despite this, the majority of the participants were enthusiastic about the online prevention course and potential bias might occur with this. It is possible that youth with negative experiences with the course or research were not willing to be interviewed. Additionally, due to the sensitivity of this problem and the fact that the parents are involved, youth might find it difficult to speak about this with a third party (ie, might feel like “airing their dirty laundry” in public). However, for both participants and providers, repetition in the interviews showed a level of saturation.

A second limitation relates to the generalizability of the findings. It is noticeable that an overwhelming majority of Kopstoring participants, participating in the underlying RCT and this process evaluation, were female. This is probably not a good reflection of an open population, assuming there are an almost equal number of boys who have a mentally ill parent as there are girls. This leaves questions about generalizability unanswered. In addition, questions have been raised such as “are the findings useful in a similar online context, but with a different underlying intervention?” and “are the findings the same when comparing the online course to a similar face-to-face group?” It appeared that several factors added up; the online aspect, age, anonymity, and sensitive problems and anonymity lead to barriers doing research within this vulnerable group. The results of this study focus on youth with parents with mental illness or addiction problems. Despite these factors, some general elements can be identified that are useful in other online settings, such as the aspect of anonymity, consent, and practical issues.

### Conclusions

Online support for offspring of parents with mental illness or addiction problems is considered effective by the participants. There are not many RCTs performed to assess the effectiveness and cost-effectiveness of online prevention programs in the field of mental health care [24]. Consequently, there are not many process evaluations of these online prevention programs performed. This hampers comparison between online programs and process of delivery and expectations. In addition, a face-to-face group is set up differently in structure and has fewer participants; therefore, it is difficult to use it in comparison to an online program. In this respect, this study is unique and sheds some light on experiences and barriers for online provision of a prevention course in the field of mental health care.

The barriers for online provision of this health intervention are minimal, but the ones that exist lie in the technical sphere. Barriers for online research are multiple and touch on different aspects, such as informed consent, anonymity, lack of time, or just lack of interest. The findings of this study may explain partly why there are substantial dropout rates when delivering online interventions. The experiences of participants and providers of the Kopstoring course give valuable insights into the process of the online provision and study of Kopstoring.

## Abbreviations

RCT: randomized controlled trial
